# Epigenetics-Based Age Acceleration Associated with 2,3,7,8 TCDD Exposure in Older Americans

**DOI:** 10.3390/ijms26041478

**Published:** 2025-02-10

**Authors:** Baek-Yong Choi, Seung-Woo Ryoo, Seok-Yoon Son, Ji-Hyeon Lee, Kyoung-Bok Min, Jin-Young Min

**Affiliations:** 1 Department of Preventive Medicine, Seoul National University College of Medicine, Seoul 03080, Republic of Korea; wdgenius@naver.com (B.-Y.C.); yeon3174@snu.ac.kr (S.-W.R.); sso1652@snu.ac.kr (S.-Y.S.); atlas928@snu.ac.kr (J.-H.L.); 2 Integrated Major in Innovative Medical Science, Seoul National University Graduate School, Seoul 03080, Republic of Korea; 3 Veterans Medical Research Institute, Veterans Health Service Medical Center, Seoul 05368, Republic of Korea

**Keywords:** environmental chemical, DNA methylation, epigenetic clocks, dioxins, general population

## Abstract

2,3,7,8-tetrachlorodibenzo-p-dioxin (TCDD) is highly toxic with potential impacts on aging. While previous studies have linked TCDD exposure to reduced telomere length and altered sperm DNA methylation (DNAm) age, its relationship with epigenetic aging remains unclear. This study investigated the association between serum TCDD levels and epigenetic clocks derived from DNAm in whole blood in older adults. Using data from the 1999–2002 National Health and Nutrition Examination Survey, we analyzed 589 participants aged 50 to 79 years with available blood TCDD and DNA methylation measures. Blood TCDD levels were measured by high-resolution gas chromatography/isotope-dilution high-resolution mass spectrometry. The six DNAm-based epigenetic clocks included Horvath Age, Hannum Age, SkinBlood Age, Pheno Age, Grim Age, and Grim Age2. Multivariable regression analysis showed significant associations between TCDD levels and Horvath Age, Hannum Age, Pheno Age, Grim Age, and Grim Age2. However, when using lipid-adjusted TCDD levels, significant associations remained only for PhenoAge (β = 0.73; SE, 0.31; *p* = 0.0258) and Grim Age2 (β = 0.44; SE, 0.21; *p* = 0.0472). The strongest non-linear trends were observed for PhenoAge, Grim Age, and Grim Age2, suggesting a threshold-dependent impact of TCDD on DNAm aging processes. Our findings suggest that TCDD exposure is associated with accelerated epigenetic aging, particularly in mortality-related clocks, with a dose-dependent and non-linear pattern.

## 1. Introduction

Dioxins are among the most toxic environmental chemicals. Small quantities of dioxins occur naturally, but they mainly originate from chemical combustion events and are inadvertently released into the environment as by-products of industrial processes, including chlorine bleaching of paper pulp and the manufacture of some herbicides and pesticides [1,2]. Dioxins are highly stable, are long-lasting, and easily bioaccumulate. Once dioxins enter the human body, they are stored in fat tissue, posing health risks to various organs and systems [3].

A particularly notorious dioxin, 2,3,7,8-tetrachlorodibenzo-p-dioxin (TCDD), is an unintentional by-product of incomplete combustion that persists in the environment and readily dissolves in fats and oils [4]. The majority of human exposure to TCDD levels is from food, particularly meat, dairy products, and fish [5]. Although serum TCDD concentrations depend on age and sex, previous studies have shown a general increase in serum TCDD levels with age in the general population [6]. TCDD exerts toxic effects on growth regulation, the endocrine system, and other factors associated with homeostasis [7,8]. Epidemiological studies have shown a significant association between TCDD exposure and a wide range of health effects [4]. Experimental animal studies have provided evidence that TCDD causes various systemic and immune dysfunctions across a wide range of exposure concentrations, including tumorigenesis, immunological dysfunction, and teratogenesis [9,10].

The aryl hydrocarbon receptor (AHR), a ligand-dependent transcription factor, mediates the toxic effects of TCDD [11]. Traditionally, the AHR is recognized as a mediator of xenobiotic metabolism in response to environmental toxins, regulating various signaling pathways related to development, detoxification, immune response, and homeostasis [12]. Previous rodent studies have demonstrated that TCDD exposure can promote the epigenetically transgenerational inheritance of adult-onset diseases [13]. A significant body of work has shown the reproductive toxicity of TCDD; for example, pregnant women may transfer a fraction of their dioxin burden to the fetus during pregnancy and to the infant via breastfeeding [14]. A recent study evaluated the relationship between dioxin exposure and sperm DNA methylation (DNAm) among Vietnam War veterans [15] and reported that serum dioxin was associated with increased sperm DNAm age. However, there is little evidence of TCDD-induced changes in DNAm in human blood.

In recent years, there has been a notable increase in the number of studies on DNAm -based measures of biological aging to better understand environmental exposure-health relationships [16]. Biological aging, measured by “epigenetic clocks”, is an individual’s degree of aging based on DNAm patterns at specific CpG sites across the genome [17]. Biological age is predictable but not strictly linked to chronological age. Accelerated biological age represents the extent to which a person’s biological age exceeds their chronological age and predicts an elevated risk of mortality and all significant age-related diseases [18]. Researchers have estimated DNAm-based epigenetic clocks; the first generation of epigenetic clocks includes the whole blood clock by Hannum et al. [19] and the pan-tissue clock by Horvath [17], which were developed using a machine learning algorithm trained to predict chronological age based on the DNAm age. Second-generation clocks, such as Pheno Age [20] and Grim Age [21,22] have been developed using clinical data to predict the risk of various aging outcomes (i.e., mortality) beyond chronological age and serve as markers of biological aging.

Based on the evidence linking DNAm to dioxin-like compound-induced AHR, exposure to TCDD has been shown to alter DNAm, resulting in accelerated epigenetic aging. However, there remains a lack of research to support this knowledge gap on the role of TCDD in epigenetic aging. Therefore, this study aimed to test the hypothesis that exposure to TCDD may alter DNAm-based epigenetic clocks. We analyzed the association between serum TCDD levels and six epigenetic clocks, including Hannum Age, Horvath Age, SkinBlood Age, PhenoAge, Grim Age, and Grim Age2.

## 2. Results

### 2.1. Study Participants’ Characteristics

Table 1 shows the characteristics of the study participants and the distribution of serum 2,3,7,8 TCDD levels across different demographic and health-related variables. A total of 589 participants were included in the study, including 295 (50.08%) females, 220 (37.35%) identified as non-Hispanic White, and 268 (45.50%) having less than a high school education. The majority of participants (386; 65.53%) reported an annual family income of $20,000 or more.

The mean (SE) TCDD of all participants was 13.43 (0.41) pg/g. Significant differences in TCDD levels were observed across age groups, sex, alcohol consumption, and hypertension status (*p* < 0.05). Participants in older age categories exhibited progressively higher TCDD levels (*p* = 0.0130), with mean levels increasing from 15.26 pg/g in the 50–59 age group to 17.43 pg/g in the 70–79 age group. Similarly, females had significantly higher TCDD levels than males (14.63 vs. 12.23 pg/g, *p* = 0.0187). For health behaviors, non-drinkers had significantly higher TCDD levels compared to drinkers (15.39 vs. 12.37 pg/g, *p* = 0.0161). Additionally, participants with hypertension had significantly higher TCDD levels than those without (14.89 vs. 12.15 pg/g, *p* = 0.0003).

### 2.2. Chronological Age and Epigenetic Age

Table 2 provides descriptions and mean (SE) values for chronological age and six DNAm-based epigenetic ages. The participants’ mean (SE) chronological age was 63.14 (0.33) years. The mean values of the six epigenetic ages differed from the chronological age and from one another. Among the epigenetic ages, Grim Age, with a mean of 63.93 (0.32) years, was the closest to the chronological age. The highest mean value was observed for Grim Age2 at 69.86 (0.32) years, while PhenoAge had the lowest mean value at 53.55 (0.41) years.

The RMSE results indicate variability in predictive performance across the clocks. Pheno Age exhibited the highest RMSE (6.64), suggesting the largest deviation from chronological age, whereas Grim Age had the lowest RMSE (3.61), indicating the closest alignment with actual age estimates. Other clocks demonstrated intermediate RMSE values, including Hannum Age (5.13), Horvath Age (4.84), SkinBlood Age (4.23), and Grim Age2 (4.16).

### 2.3. TCDD Exposure and Epigenetic Age Acceleration

Table 3 shows the regression analysis results for the association between epigenetic age and 2,3,7,8 TCDD. In the unadjusted model, a one-unit increase in TCDD was associated with an increase in Horvath Age, Hannum Age, PhenoAge, Grim Age, and Grim Age2. The observed association remained after adjusting for age, sex, educational levels, family income, cigarette smoking, alcohol drinking, moderate activity, obesity, and comorbidities. Multivariable regression analysis revealed a significant association of Horvath Age (β = 0.06; SE, 0.02; *p* = 0.0059), Hannum Age (β = 0.07; SE, 0.03; *p* = 0.0345), PhenoAge (β = 0.11; SE, 0.04; *p* = 0.0166), Grim Age (β = 0.06; SE, 0.02; *p* = 0.0212), and Grim Age2 (β = 0.07; SE, 0.03; *p* = 0.0210). That means an increase in TCDD exposure by one unit was associated with a 0.06-year (approximately 22 days) acceleration in epigenetic age as measured by HorvathAge. Similarly, a one-unit increase in TCDD exposure was linked to an acceleration ranging from 0.06 to 0.11 years (approximately 26–40 days) in Hannum Age, PhenoAge, Grim Age, and Grim Age2.

**Figure 1:**
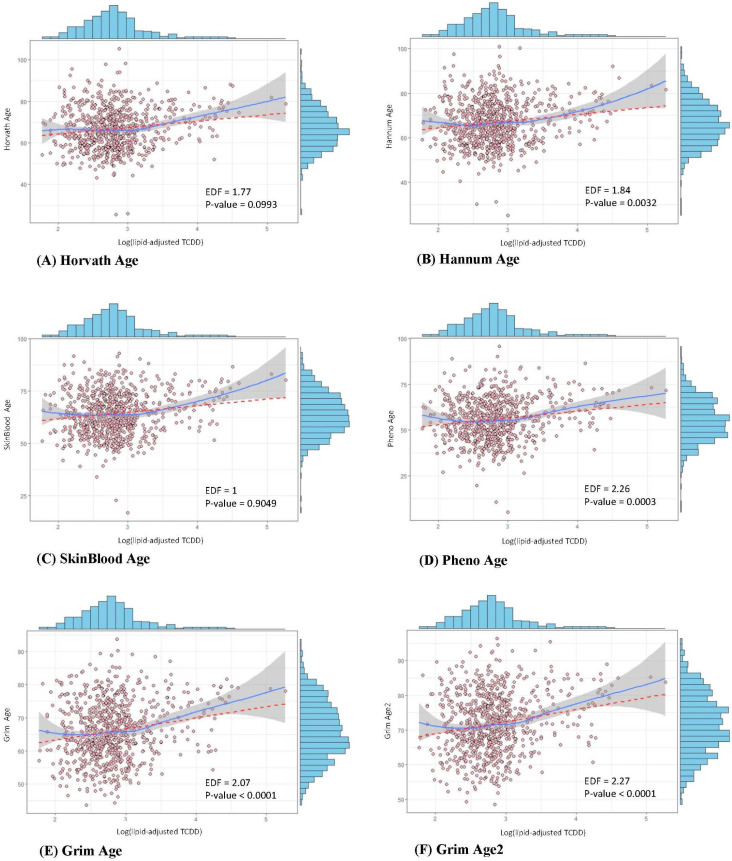
Generalized Additive Model (GAM) analysis of the relationship between TCDD exposure and epigenetic aging clocks: (**A**) Horvath Age, (**B**) Hannum Age, (**C**) SkinBlood Age, (**D**) PhenoAge, (**E**) Grim Age, and (**F**) Grim Age2. Each pink dot in the scatter plots represents an observed data point. The red dashed line represents the linear regression fit, while the blue solid line depicts the non-linear regression fit, and the grey shaded area represents the 95% confidence interval (CI). Histograms positioned above and to the right of each main plot illustrate the marginal distributions of TCDD levels and epigenetic age measures, respectively. All models were adjusted for age, sex, race/ethnicity, education, family income, smoking, alcohol consumption, physical activity, obesity, and comorbidities.

Figure 1 illustrates the relationship between log-transformed lipid-adjusted TCDD levels and six epigenetic aging measures, analyzed using GAM. The EDF values provide insight into the nature of these relationships. An EDF value of 1 suggests a linear association, whereas values between 1 and 2 indicate mild non-linearity, and values greater than 2 denote a strong non-linear relationship. Our data showed that PhenoAge (EDF = 2.26), Grim Age (EDF = 2.07), and Grim Age2 (EDF = 2.27) exhibit the highest degree of non-linearity, with epigenetic aging acceleration becoming more pronounced at higher TCDD exposure levels.

In Table 4, lipid-adjusted values of serum TCDD provide a more accurate picture of the total burden of these residues in fatty tissue throughout the body. We further analyzed the association between epigenetic age and lipid-adjusted TCDD levels. In contrast to the association using serum TCDD (Table 3), lipid-adjusted 2,3,7,8 TCDD levels were significantly associated with PhenoAge (β = 0.73; SE, 0.31; *p* = 0.0258) and Grim Age2 (β = 0.44; SE, 0.21; *p* = 0.0472), but not Horvath Age, Hannum Age, and Grim Age. This result indicates that a one-unit increase in lipid-adjusted TCDD exposure corresponds to a 0.73-year (approximately 8.8 months) acceleration in PhenoAge and a 0.44-year (approximately 5.3 months) acceleration in Grim Age2, highlighting the potential biological impact of TCDD exposure.

## 3. Discussion

### 3.1. Key Points of Results

This study examined whether exposure to TCDD was associated with accelerated epigenetic aging in the general population. Higher serum TCDD levels were significantly associated with advanced epigenetic aging, particularly in Horvath Age, Hannum Age, Pheno Age, Grim Age, and Grim Age2. However, when lipid-adjusted TCDD levels were analyzed, only Pheno Age and Grim Age2 remained significantly associated. This difference suggests that TCDD may specifically influence aging processes linked to inflammation and metabolic health, pathways captured by Pheno Age and Grim Age2. These findings highlight the potential of TCDD exposure to modulate DNAm-based epigenetic aging, particularly through lipid-adjusted metrics that better account for serum lipid variability.

Notably, our findings indicate a non-linear association between TCDD exposure and epigenetic aging across multiple epigenetic clocks. While lower to moderate TCDD levels showed relatively weak associations, the relationship became more pronounced at higher exposure levels, particularly in Hannum Age, Pheno Age, Grim Age, and Grim Age2, where epigenetic aging acceleration was more evident. This pattern suggests a dose-dependent effect, with a potential threshold beyond which TCDD exposure exerts a stronger influence on biological aging markers. Such non-linearity reflects the complex interplay between TCDD bioaccumulation, DNAm dynamics, and aging processes.

The pronounced elevation of TCDD levels in the 70–79 age group may underscore the cumulative nature of exposure and its potential long-term impact on health. Older individuals may retain higher levels of lipophilic toxicants due to prolonged exposure and slower clearance rates, as noted in previous studies [23]. Longitudinal studies are also necessary to disentangle the temporal dynamics of TCDD accumulation and its causal relationship with accelerated biological aging.

To the best of our knowledge, only one previous study has directly examined the association between dioxin exposure and epigenetic aging. The study reported a significant association between serum dioxin levels and sperm DNAm age. Specifically, a 1% increase in serum dioxin was significantly associated with a 0.0126-year increase in JenkinsAge and a 0.0130-year increase in HorvathAge. A prior study also found a significant association between TCDD exposure and HorvathAge [15]; however, we did not observe this association in our lipid-adjusted model. This discrepancy may be attributed to the differences in sample types, as the previous study analyzed sperm samples, whereas our study utilized serum samples. When comparing these results to age acceleration observed in sperm samples, it is essential to recognize the key differences between tissues. Whole blood DNA reflects systemic epigenetic age acceleration, whereas sperm DNA represents tissue-specific epigenetic changes that may vary due to differences in cell turnover rates, environmental exposures, and biological functions. Therefore, caution must be exercized when generalizing findings from whole blood to sperm or other tissue types.

Despite this discrepancy, the overall trend between dioxin exposure and epigenetic aging remains consistent with previous findings. Although there is no direct linking of dioxin exposure to epigenetic aging, studies have investigated its relationship with another biological aging marker: telomere length. A systematic review has demonstrated a relationship between age and leukocyte telomere length (LTL) in adults. A cross-sectional study found a significant negative correlation between age and mean LTL [24]. Moreover, some studies have reported a negative association between epigenetic aging acceleration and relative LTL [25,26]. Additionally, exposure to persistent organic pollutants, including dioxins, has been associated with longer LTL in US adults, based on NHANES data [27].

### 3.2. Detail of Mechanism

The biological mechanisms through which TCDD exposure affects epigenetic aging remain unclear, but several hypotheses have been proposed. TCDD’s toxicity is primarily mediated by its action as a ligand of the aryl hydrocarbon receptor (AHR), which promotes the expression of inflammatory markers and elevates oxidative stress [28]. TCDD-mediated inflammation is triggered by an increase in intracellular calcium, activation of phospholipase A2, and cyclooxygenase-2 (COX-2) enzymes that play a key role in the production of inflammatory prostaglandins. Notably, COX-2 is a direct target of AHR [29].

In addition to inflammation, oxidative stress and DNA repair mechanisms play a crucial role in the biological effects of TCDD exposure. TCDD induces reactive oxygen species (ROS), causing oxidative stress and subsequent damage to lipids, proteins, and DNA [30]. Biomarkers of TCDD-induced oxidative stress include the production of superoxide anions, lipid peroxidation, and DNA single-strand breaks. This oxidative stress has been directly linked to carcinogenesis and accelerated biological aging [31]. For example, Lin et al. (2007) demonstrated that TCDD exposure leads to increased reactive oxygen species (ROS) formation, depletion of intracellular glutathione, and DNA strand breaks in human breast cancer cell lines [32]. The authors suggested that the TCDD-induced oxidative stress and DNA damage contribute to TCDD-induced carcinogenesis. Chan et al. (2004) reported that TCDD exposure impairs the repair of DNA double-strand breaks through homologous recombination [33]. This impairment can contribute to genomic instability and carcinogenesis. Korkalainen et al. (2012) found that TCDD induced delayed genomic instability associated with impaired DNA damage response mechanisms [34]. These delayed effects, alongside TCDD’s interaction with ROS, may contribute to its carcinogenic potential.

Furthermore, TCDD’s activation of AHR modulates the expression of genes involved in oxidative stress responses and DNA repair pathways, such as base excision repair and nucleotide excision repair. However, chronic TCDD exposure may dysregulate these pathways, contributing to sustained DNA damage, aging acceleration, and increased mortality risks [35]. TCDD, as an AHR agonist, induces over-activation of AHR expression. Unlike natural ligands such as quercetin and curcumin, TCDD produces free radicals that exacerbate harmful effects on aging and DNA integrity. For example, polycyclic aromatic hydrocarbons (PAHs), which act as ligands for the AHR similar to dioxin [36], have also been reported to accelerate methylation aging among healthy Chinese participants [37]. Similarly, a study on coke-oven workers, who are exposed to PAHs, reported that the proportion of individuals with biological aging older than chronological age was statistically significantly higher compared to the control group [38].

In this study, TCDD exposure was found to have statistically significant associations with epigenetic aging, particularly in the Pheno Age and Grim Age2 models. The PhenoAge model, which includes inflammatory markers such as C-reactive protein (CRP) and alkaline phosphatase (ALP), showed a strong association with TCDD exposure. Although the Grim Age model showed marginal significance (*p*-value of 0.0526), the Grim Age2 model exhibited a statistically significant association, which includes CRP and ALP, demonstrating a stronger link to inflammation. These findings align with the hypothesis that TCDD induces both inflammation and oxidative stress, which together accelerate aging.

### 3.3. Strength and Limitations

The primary strength of our study is its basis in the NHANES dataset, which includes comprehensive information on demographics, socioeconomic status, and health conditions. The NHANES data are instrumental in biomonitoring environmental exposures to toxic chemicals and generating national estimates of disease prevalence. The NHANES procedures and measures are strictly confidential and may warrant the reliability of estimates as study results.

However, our study also has several limitations. We relied on a single measurement of dioxin levels in serum, which may not accurately reflect lifetime exposure. Despite this, considering the long half-life of dioxin in serum (approximately seven years or more) [39] and assuming relatively constant environmental exposure sources over time, we posited that serum dioxin levels in a given participant would remain consistent over the years. Additionally, we cannot rule out the possibility of residual confounding from variables not measured in this study, even though our statistical models accounted for the known risk factors of epigenetic aging acceleration [40]. While TCDD exposure contributes to epigenetic aging, various sociodemographic, lifestyle, and metabolic factors also play significant roles. In our study, we adjusted for smoking, alcohol consumption, obesity, metabolic conditions (diabetes, hypertension, and dyslipidemia), and educational attainment, which are well-established contributors to biological aging. However, socioeconomic status (SES) encompasses more than just education and family income and may still confound the observed association between TCDD and epigenetic aging. SES has been linked to increased environmental pollutant exposure and accelerated aging through mechanisms involving chronic stress, poor diet, and limited healthcare access [41,42]. Nearly half of the participants in this study had an education level below high school, suggesting lower SES, which may have a greater impact on biological aging than TCDD exposure. Future research should further investigate the complex interplay between environmental, behavioral, and biological determinants of epigenetic aging, with a particular focus on SES beyond educational attainment and other unmeasured risk factors. Furthermore, potential biases due to misclassification of self-reported data and uncontrolled confounding by unmeasured factors, particularly other environmental exposures that pose a high risk of dioxin contamination, were not addressed. In summary, improving methods in terms of dioxin exposure uncertainty and unmeasured confounding variables should be priorities for future research. Such improvements would enhance our understanding of the role of TCDD exposure in epigenetic aging and help identify critical mechanisms underlying this relationship.

## 4. Methods

### 4.1. Study Population

The data were obtained from the 1999–2000 and 2001–2002 cycles of the National Health and Nutrition Examination Survey (NHANES), during which blood for DNA purifications was collected. NHANES, conducted by the Centers for Disease Control and Prevention (CDC), is a representative national survey of the non-institutionalized civilian population in the United States. The study protocols were approved by the National Center for Health Statistics Institutional Review Board (https://www.cdc.gov/nchs/nhanes/irba98.htm (accessed on 15 December 2024)). In addition, all participants provided verbal and written consent for future research.

We merged two NHANES cycles (1999–2000 and 2001–2002), resulting in a total of 21,004 participants. From this group, we initially selected 2191 individuals aged 50 to 79 years for whom DNAm analyses had been completed. Among these, 665 participants had available TCDD measurements. The final study population included 589 individuals with no missing data for the following covariates: age, sex, ethnicity, education, annual family income, smoking, drinking status, obesity, physical activity, and history of diseases, including hypertension, diabetes, and dyslipidemia.

### 4.2. DNA Methylation Profiling and Determination of Epigenetic Age

DNA was extracted from whole blood, and specimens were stored at −80 °C. The DNAm assay was performed at Duke University. The bisulfite conversion of DNA was carried out according to the manufacturer’s instructions. Specifically, 500 ng of DNA was bisulfite treated using a Zymo EZ DNA Methylation kit (cat# D5001, Zymo Research, Irvine, CA, USA) under PCR conditions optimized for Illumina’s Infinium Methylation assay (95 °C for 30 s, 50 °C for 60 min × 16 cycles).

Data were generated using the Illumina Infinium MethylationEPIC BeadChip v1.0 (cat# WG317-1001, Illumina, San Diego, CA, USA). A total of 4 μL of bisulfite-converted DNA was hybridized to the Illumina BeadChip following the manufacturer’s protocol. The samples were denatured and amplified overnight (20–24 h). After incubation, fragmentation, precipitation, and resuspension were performed prior to hybridization with the EPIC BeadChip (16–24 h). The BeadChip was then washed to remove any unhybridized DNA and labeled with nucleotides to extend the primers to the DNA. Following the Infinium HD Methylation protocol, the BeadChip was imaged using the Illumina iScan system (Illumina, San Diego, CA, USA).

For quality control, we identified probes within each epigenetic biomarker that were missing in more than 5% of samples. Imputation was conducted in two different ways based on the recommendations of the DNAm epigenetic biomarker creators. For Horvath, Hannum, SkinBlood, PhenoAge, Grim Age, and Grim Age2, missing DNAm beta values were imputed using a gold standard reference dataset developed by Horvath, where the mean DNAm beta values were substituted for missing data points at each DNAm site.

Detailed information regarding the development and methodology of epigenetic biomarkers is available on the CDC website, which provides comprehensive guidance on data profiling, biomarker production, and analytic methodologies (https://wwwn.cdc.gov/nchs/nhanes/ (accessed on 15 December 2024)).

### 4.3. Serum TCDD Concentrations

Whole blood and urine samples were collected at the Mobile Examination Centers, processed, and stored under appropriate frozen conditions (−20 °C) until they were shipped to the National Center for Environmental Health for analysis, following the procedures outlined in the Laboratory Procedures Manual.

Serum TCDD concentrations were measured using high-resolution gas chromatography/isotope-dilution high-resolution mass spectrometry (HRGC/ID-HRMS). Serum samples were spiked with ^13^C_12_-labeled internal standards, and the analytes of interest were isolated using either a C18 solid-phase extraction or liquid–liquid extraction procedure, followed by a multi-column automated cleanup and enrichment procedure.

The analytes were chromatographed on a DB-5 ms capillary column (30 m × 0.25 mm × 0.25 μm film thickness) on a Hewlett-Packard 6890 gas chromatograph. Selected analytes were quantified by ID-HRMS using selected ion monitoring at 10,000 resolving power with either a Micromass AutoSpec ULTIMA or Finnigan MAT95 mass spectrometer operating in the electron ionization mode. The concentration of each analyte was determined using an individual standard linear calibration. Each analytical run was conducted in a blinded manner and included three unknown serum samples, a method blank, and a quality control sample to ensure accuracy and reliability.

Lipid adjustment was applied to account for inter-individual variability in serum lipid levels, ensuring the measured associations reflect the true biological effects of TCDD. Lipid-standardized concentrations (pg/g lipid) were calculated as TCDD levels multiplied by total lipid (total lipid = 2.27 × total cholesterol + triglyceride + 62.3). This approach, widely used in studies of lipophilic contaminants [43,44], minimizes confounding from lipid variability.

The lower limits of detection (LLOD) for TCDD were calculated at three times the estimated standard deviation at zero analyte concentration [45]. For analytes with values below the LLOD, the results field was populated with an imputed value equal to the LLOD divided by the square root of 2 (LLOD/√2).

### 4.4. Variables of Interest

Covariates were selected from questionnaire data on demographics, socioeconomic status, health behaviors, and health conditions based on clinical importance and univariate analysis [46]. The selected covariates included age, sex, ethnicity, education, family income, smoking, alcohol use, physical activity, obesity, diabetes mellitus, hypertension, and dyslipidemia.

Sociodemographic variables included age, sex (male or female), ethnicity (white, black, Hispanic, or others), education (no high school diploma, high school diploma only, or college diploma), and annual family income (less than $20,000 or $20,000 or more).

Health-related behavior variables included smoking status (current smoker, ex-smoker, or never smoker), alcohol drinking (drinker or non-drinker), physical activity (yes or no), and body mass index (BMI). For smoking status, ex-smokers and never smokers were classified based on whether they had smoked at least 100 cigarettes in their lifetime. Participants who consumed at least 12 alcoholic beverages per year (equivalent to 12 oz. of beer, a 5 oz. glass of wine, or 1.5 oz. of liquor) were categorized as drinkers [47]. Physical activity status was identified as engaging in moderate-intensity activity (activities that cause a significant increase in breathing or heart rate) for at least 10 min per session, at least two days per week. BMI was calculated as weight (kg) divided by height (m^2^), and participants was categorized into two groups based on BMI: non-obese (<30 kg/m^2^) and obese (≥30 kg/m^2^).

Health conditions included a history of diabetes (yes or no), hypertension (yes or no), and dyslipidemia (yes or no).

### 4.5. Statistical Analysis

The mean and standard errors (SE) of 2,3,7,8-TCDD levels were calculated based on the characteristics of the study population. To assess the predictive accuracy of epigenetic clocks in estimating chronological age, Root Mean Square Error (RMSE) values were computed for each clock.

The association between epigenetic age (dependent variable) and 2,3,7,8-TCDD levels (independent variable, both lipid-adjusted and non-lipid-adjusted) was evaluated using multivariable linear regression. Beta coefficients (SE) were estimated to quantify these associations. The regression models were adjusted for age, sex, ethnicity, education, smoking status, alcohol consumption, BMI, physical activity, hypertension, diabetes, and dyslipidemia. To explore potential non-linear relationships, Generalized Additive Models (GAM) were employed. The GAM plots include the estimated degrees of freedom (EDF) and *p*-values, which provide insight into the non-linearity of each epigenetic clock. An EDF of 1 indicates a linear relationship, values between 1 and 2 suggest mild non-linearity, and values greater than 2 denote a strong non-linear association.

All statistical analyses incorporated weighted estimates of population parameters in accordance with the NHANES Analytic and Reporting Guidelines. Statistical analyses were conducted using SAS 9.4 (SAS Institute, Cary, NC, USA) and R version 4.4.2. A statistical significance threshold of α = 0.05 was applied.

## 5. Conclusions

In conclusion, exposure to TCDD was associated with epigenetic aging, with stronger associations observed in the “second-generation” clocks of Pheno Age and Grim Age2. Notably, our analysis revealed a non-linear relationship, where the association between TCDD levels and epigenetic aging became more pronounced at higher exposure levels, suggesting a potential threshold effect. Our findings indicate that TCDD exposure may accelerate epigenetic aging, particularly in mortality-related clocks, underscoring its potential role in aging and aging-related diseases.

Compared with well-established risk factors of epigenetic age acceleration, such as smoking, obesity, and chronic inflammation, the impact of TCDD exposure is relatively smaller. However, it remains biologically relevant, particularly due to its persistent and cumulative nature over time. While TCDD is not the most potent aging accelerator, our findings suggest that long-term, low-dose exposure may contribute to biological aging in a manner similar to other environmental risk factors. Future research is needed to replicate these findings in diverse populations and to investigate the long-term effects of cumulative TCDD exposures on epigenetic aging.

## Figures and Tables

**Table 1:** Participants’ characteristics and distribution of serum 2,3,7,8 TCDD (*n* = 589).

Characteristics	N	(%)	2,3,7,8-TCDD (pg/g)		*p*-Value
			Mean	(SE)	
Age (years)					0.0130
50–59	196	(29.21)	15.26	(0.40)	
60–69	242	(36.07)	15.90	(0.43)	
70–79	151	(22.50)	17.43	(0.71)	
Sex					0.0187
Male	294	(49.92)	12.23	(0.38)	
Female	295	(50.08)	14.63	(0.72)	
Race/ethnicity					0.1584
Non-Hispanic White	220	(37.35)	12.32	(0.57)	
Non-Hispanic Black	121	(20.54)	16.51	(1.33)	
Hispanic	225	(38.2)	12.81	(0.53)	
Others	23	(3.9)	14.07	(1.69)	
Educational level					0.0561
Less than high school	268	(45.50)	14.01	(0.63)	
High school	108	(18.34)	12.47	(0.75)	
Above high school	213	(36.16)	13.20	(0.72)	
Annual family income					0.6591
Less than $20,000	203	(34.47)	13.70	(0.76)	
$20,000 or more	386	(65.53)	13.30	(0.48)	
Cigarette smoking					0.3354
Current smoker	101	(17.15)	13.55	(0.91)	
Past smokers	220	(37.35)	12.59	(0.51)	
Never smoked	268	(45.50)	14.09	(0.72)	
Alcohol drinking					0.0161
Yes	381	(64.69)	12.37	(0.33)	
No	208	(35.31)	15.39	(0.98)	
Moderate activity					0.1237
Yes	219	(37.18)	13.03	(0.59)	
No	343	(58.23)	13.56	(0.57)	
Unable	27	(4.58)	15.04	(2.00)	
Obesity (BMI ≥ 30 kg/m^2^)					0.1116
No	378	(64.18)	12.56	(0.42)	
Yes	211	(35.82)	14.99	(0.86)	
Diabetes					0.2546
Yes	112	(19.02)	15.18	(1.30)	
No	477	(80.98)	13.02	(0.40)	
Hypertension					0.0003
Yes	276	(46.86)	14.89	(0.73)	
No	313	(53.14)	12.15	(0.41)	
Dyslipidemia					0.2158
Yes	292	(49.58)	14.13	(0.64)	
No	297	(50.42)	12.75	(0.52)	

TCDD, Tetrachlorodibenzodioxin; BMI, body mass index (kg/m^2^); *p*-values indicate the statistical significance of differences in serum TCDD levels across participant characteristics, with values less than 0.05 considered statistically significant.

**Table 2:** Chronologic age and DNA methylation-based epigenetic age.

Variables	Description	Mean	(SE)	RMSE
Age	Chronologic age	63.14	(0.33)	-
HorvathAge	Horvath DNA methylation predicted chronological age in 51 tissues	64.79	(0.34)	4.84
HannumAge	Hannum DNA methylation predicted chronological age in whole blood	64.68	(0.35)	5.13
SkinBloodAge	Horvath DNA methylation predicted chronological age in skin and blood derived tissues	62.00	(0.35)	4.23
PhenoAge	Levine DNA methylation predicted phenotypic age in whole blood	53.55	(0.41)	6.64
Grim Age	Horvath DNA methylation predicted mortality in whole blood	63.93	(0.32)	3.61
GrimAge2	Horvath updated DNA methylation predicted mortality in whole blood	69.86	(0.32)	4.16

**Table 3:** Results of multivariable regression analysis for association between epigenetic age and 2,3,7,8 TCDD exposure.

Epigenetic Age	Unadjusted Model			Adjusted Model								
				Model 1			Model 2			Model 3		
	β	(SE)	*p*-Value	β	(SE)	*p*-Value	β	(SE)	*p*-Value	β	(SE)	*p*-Value
HorvathAge	0.16	(0.04)	0.0003	0.05	(0.02)	0.0123	0.05	(0.02)	0.0130	0.06	(0.02)	0.0059
HannumAge	0.16	(0.04)	0.0002	0.05	(0.03)	0.0655	0.06	(0.03)	0.0505	0.07	(0.03)	0.0345
SkinBloodAge	0.14	(0.04)	0.0010	0.01	(0.02)	0.4934	0.02	(0.02)	0.4115	0.02	(0.02)	0.2401
PhenotypicAge	0.23	(0.06)	0.0006	0.10	(0.04)	0.0310	0.11	(0.05)	0.0316	0.11	(0.04)	0.0166
GrimAge	0.16	(0.04)	0.0003	0.06	(0.03)	0.0376	0.06	(0.03)	0.0363	0.06	(0.02)	0.0212
GrimAge2	0.17	(0.04)	0.0002	0.07	(0.03)	0.0383	0.07	(0.03)	0.0391	0.07	(0.03)	0.0210

β, beta coefficient; SE, standard error; Model 1 = Adjusted for age, sex, and race/ethnicity; Model 2 = Model 1 + educational level and family income; Model 3 = Model 2 + smoking, alcohol drinking, moderate physical activity, obesity, and comorbidities.

**Table 4:** Results of multivariable regression analysis for association between epigenetic age and lipid-adjusted values of 2,3,7,8 TCDD levels.

Epigenetic Age	Unadjusted Model			Adjusted Model								
				Model 1			Model 2			Model 3		
	β	(SE)	*p*-Value	β	(SE)	*p*-Value	β	(SE)	*p*-Value	β	(SE)	*p*-Value
HorvathAge	0.99	(0.34)	0.0072	0.24	(0.16)	0.1533	0.25	(0.17)	0.1426	0.28	(0.16)	0.1003
HannumAge	1.03	(0.32)	0.0028	0.35	(0.22)	0.1238	0.40	(0.23)	0.0988	0.42	(0.23)	0.0736
SkinBloodAge	1.00	(0.33)	0.0048	0.16	(0.14)	0.2470	0.19	(0.14)	0.1984	0.22	(0.14)	0.1234
PhenotypicAge	1.47	(0.37)	0.0005	0.66	(0.32)	0.0486	0.70	(0.34)	0.0471	0.73	(0.31)	0.0258
GrimAge	1.02	(0.31)	0.0024	0.28	(0.21)	0.1984	0.31	(0.22)	0.1779	0.36	(0.18)	0.0526
GrimAge2	1.11	(0.30)	0.0010	0.38	(0.26)	0.1549	0.41	(0.27)	0.1417	0.44	(0.21)	0.0472

β, beta coefficient; SE, standard error; Model 1 = Adjusted for age, sex, and race/ethnicity; Model 2 = Model 1 + educational level and family income; Model 3 = Model 2 + smoking, alcohol drinking, moderate physical activity, obesity, and comorbidities.

## Data Availability

The data was retrieved from publicly available resources and can be accessed from National Center for Health Statistics of Center for Disease Control and Prevention through https:/www.cdc.gov/nchs/nhanes/index.htm (accessed on 15 December 2024).
